# A Novel BiOBr/CAU‐17 Composite with Enhanced Photo‐Catalytic Performance for Dye Degradation and Removal of Tetracycline Antibiotic Under Visible Light

**DOI:** 10.1002/open.202400195

**Published:** 2024-10-23

**Authors:** Mansoor Akhtar, Shifa Ullah Khan, Ghulam Mustafa, Muhammad Ahmad, Tansir Ahamad

**Affiliations:** ^1^ College of Civil and Transportation Engineering Shenzhen University Shenzhen, Guangdong 518060 P. R. China; ^2^ Institute for Advanced Study Shenzhen University Shenzhen, Guangdong 518060 P. R. China; ^3^ The Institute of Chemistry Faculty of Science University of Okara Renala Campus 56100 Punjab Pakistan; ^4^ Department of Chemistry College of Science King Saud University Riyadh 11451 Saudi Arabia

**Keywords:** Photocatalytic degradation, MOFs, BiOBr/CAU-17, Rhodamine B (RhB), Nanocomposites

## Abstract

In order to improve the low specific surface area and high recombinant light generation carriers of BiOBr, loading BiOBr onto suitable Metal Organic Frameworks (MOFs) is an effective strategy to unleash its efficient visible light response and intrinsic catalytic activity. In this study, using classic MOF CAU‐17 as a precursor, using a straightforward co‐precipitation technique, four BiOBr/CAU‐17 composites with distinct MOF contents values BCAU‐1, BCAU‐2, BC, AU‐3, and BCAU‐4 were created, and their photo‐catalytic characteristics were examined. The BCAU‐2 composite exhibited much higher photo‐catalytic degradation efficiency for Rhodamine B (RhB) and Tetracycline (TC) than the pristine materials, counter compositions, and early reported materials. XRD, SEM, TEM, XPS, and EDX results revealed the strong synergistic photo‐catalytic effect of BiOBr and CAU‐17. The photocatalytic degradation of TC was significantly enhanced by the BiOBr bimetal modification, with the 2 wt.% BiOBr/CAU‐17 nanocomposite achieving an 87.2 % degradation of TC and 82 % Total Organic Carbon (TOC) removal within 60 min. The high photo‐degradation efficiency of BCAU‐2 composite should be attributed to the efficient transfer of photo‐generated carriers at interfaces and the synergistic effect between BiOBr/CAU‐17. Furthermore, the experiments on the capture of the active species proved that the main active free radicals involved in the degradation of RhB and TC are attributed to the photo‐induced holes h^+^ and ⋅ O_2_^−^ under visible light. The catalyst's efficacy is corroborated by the outcomes of photoluminescence spectroscopy and photo current response. This study offers a new understanding for the design of green synthesis schemes for photo‐catalytic dye degradation and removal of certain antibiotics from the aquatic environment.

## Graphical abstract

The BiOBr/CAU‐17 composite exhibited much higher photo‐catalytic degradation efficiency for Rhodamine B (RhB) and Tetracycline (TC) than the pristine materials, counter compositions, and early reported materials. These results revealed the strong photo‐catalytic effect of BiOBr and CAU‐17. The effectiveness of the catalyst is further verified by the results of photo current response and photo‐luminescence spectroscopy.

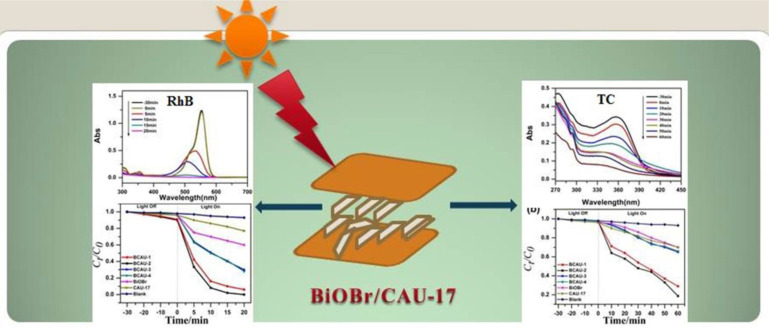

## Introduction

Visible light promoted photo‐catalysis of semiconductors has garnered incredible attention due to their wide spread application in environmental pollutant remediation.[^1^, ^2^] A prerequisite for the development of semi‐conductor photo‐catalysis should have an extended spectral responsive range and low reunion probability of photo generated holes and electrons.^[3]^ So far, various semiconductor materials, such as metallic oxides,[^4^, ^5^] nitrides,^[6]^ and sulfide have been extensively studied as photo‐catalysts.[^7^, ^8^] Among them, oxygen halogenated bismuth (BiOX, X=Cl, Br, I), as a popular semiconductor class, has been extensively implemented in the field of photo‐catalytic organic pollutant degradation.[^9^, ^10^, ^11^, ^12^, ^13^, ^14^, ^15^, ^16^, ^17^, ^18^, ^19^] Among these BiOX catalysts, BiOBr has excellent catalytic properties and has been widely explored due to its efficient visible light response. It possesses good chemical stability, unique layered structure, low cost, and excellent photo‐catalytic activity.[^20^, ^21^, ^22^, ^23^, ^24^, ^25^, ^26^, ^27^, ^28^, ^29^] However, the photo‐catalytic activity of pure BiOBr has been limited due to its relatively low specific surface area and high recombinant light generation carriers resulting low performance. Therefore, improving its photo‐catalytic performance is still a huge challenge and high requirement for the practical application in the future.[^27^, ^28^] On similar lines, the Metal Organic Frameworks (MOFs) with tunable pore channels, structural diversity and the high specific surface area make it valuable candidate in material chemistry over the past decade.[^29^, ^30^, ^31^, ^32^, ^33^, ^34^, ^35^, ^36^, ^37^]

In recent years, MOFs has been able to effectively reduce the status of organic pollutants.[^38^, ^39^, ^40^, ^41^, ^42^, ^43^, ^44^, ^45^, ^46^] In addition, MOFs with a rich accessible surface area helps to increase the load and uniform dispersion of semiconductors, thereby ultimately increasing catalytic activity.^[47]^ Recently, some researchers have reported a range of BiOBr/MOFs composites with good photo‐catalytic performance such as BiOBr/NH_2_‐MIL125 (Ti),^[48]^ BiOBr/UiO‐66‐NH_2_,^[49]^ BiOBr/UiO‐66(Zr),[^50^, ^51^, ^52^, ^53^, ^54^, ^55^] BiOBr/MOF‐5^[43]^ and so on. Therefore, it is particularly important to select the appropriate MOFs as the substrate to load semiconductor materials. Bi‐MOFs picking up great concern because of non‐toxic, water stability and possess permanent porosity.

The heterojunction formed between BiOBr and CAU‐17 facilitates the separation of photogenerated electron‐hole pairs, thereby reducing the likelihood of recombination, a common issue in photocatalysis that limits efficiency.[^56^, ^57^, ^58^] The intimate contact between BiOBr and CAU‐17 in the composite leads to a synergistic effect, where the photocatalytic performance of the composite is greater than the sum of its individual components. Dyes (e. g., methylene blue, rhodamine B), medications, and pesticides can be degraded using the BiOBr/CAU‐17 composite under visible light irradiation. The photocatalytic efficacy of the BiOBr/CAU‐17 composite in the degradation of organic dyes under visible light was investigated.^[48]^ Utilizing a solvothermal technique, BiOBr and CAU‐17 were combined to create the composite. Tests of the photocatalytic activity were conducted using dyes like methylene blue and rhodamine B. The composite of BiOBr and CAU‐17 exhibits a noteworthy enhancement in photocatalytic performance because of its expanded active sites, effective charge separation, and improved light absorption.^[51]^ Because of these characteristics, it is a very useful material for environmental applications, especially when it comes to the degradation of pollutants in the presence of visible light. This composite could be further optimized for a variety of industrial and environmental applications with future research and development.^[50]^

In this study, the BiOBr/CAU‐17 composites were synthesized via co‐precipitation from BiOBr and CAU‐17 MOF, and their photo‐catalytic properties were investigated. Photo‐catalytic results show that these new BiOBr/CAU‐17 photo‐catalysts have significantly improved visible light catalytic response towards RhB and Tetracycline degradation. The possible photo‐catalytic mechanism of RhB dye and TC degradation over BiOBr/CAU‐17 was studied in detail. The XRD patterns of BiOBr, Bi‐MOFs (CAU‐17), and the BCAU‐2 composite are very well matched, indicating that the composite of BiOBr/Bi‐MOFs is successfully prepared, and the structure of MOFs is maintained. The BCAU‐2 composite exhibited much higher photo‐catalytic degradation efficiency for RhB and TC than the pristine materials, counter compositions, and early reported materials. The SEM and TEM images showed that the BCAU‐2 composite has a smaller particle size and a more uniform distribution than BiOBr and CAU‐17. The XPS results revealed that BiOBr in the composite had a higher binding energy than that in pure BiOBr, indicating that the electron density around Bi atoms increased after the composite formed. The EDX mapping showed the uniform distribution of Bi, O, and Br elements in the composite. The experiments on the capture of the active species proved that the main active free radicals involved in the degradation of RhB and TC are attributed to the photo.^[41]^

## Results and Discussions

**Figure 1:**
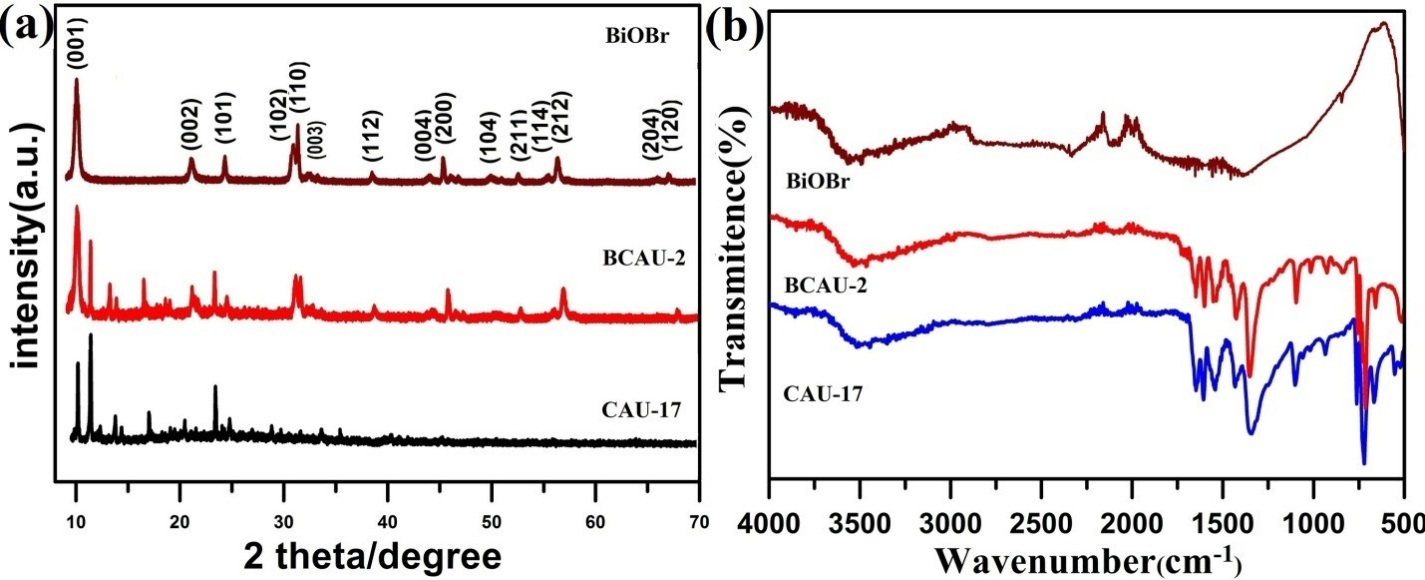
(a) The XRD patterns and (b) FT‐IR spectra of CAU‐17, BCAU‐2 and BiOBr.

As shown in Figure 1a, the XRD patterns of BiOBr, Bi‐MOFs (CAU‐17) and the BCAU‐2 composite are very well matched. The diffraction peaks at 10.88°, 21.90°, 25.13°, 31.72° 32.20°, 39.39°, 45.00°, 46.21°, 57.23° could be assigned to BiOBr (JCPDS: 73–2061). All the peaks of Bi‐MOFs (CAU‐17) are in good agreement with the literature and indicate the successful preparation of Bi‐MOFs.^[44]^ After the co‐precipitation of BiOBr and Bi‐MOFs, both of the diffraction peaks of BiOBr and Bi‐MOFs can be observed in the XRD pattern of BCAU‐2 composite. This result indicates that the composite of BiOBr/Bi‐MOFs is successfully prepared. It also suggests that the structure of MOFs is maintained. Similar results can be found in the literature.[^41^, ^42^] The XRD results of other composites are given in Figure S1.

FT‐IR results are shown in Figure 1b. The IR spectrum of BiOBr is shown in the brown curve. The peak at 511 cm^−1^ is related to Bi−O stretching vibrations. A broad band at 3420 cm^−1^ is related to the O−H bending vibration of adsorbed H_2_O molecules.^[45]^ CAU‐17 MOFs spectrum consists of peaks at 1370–1620 cm^−1^, which attributed to carboxyl group. The sharp peaks at 500–800 cm‐1 were designated to the vibrations of O−Bi−O groups. Moreover, the stretch modes of MOFs and BiOBr also can be found in BCAU‐2. As compared with the CAU‐17, the peak position of BCAU‐2 moves slightly to the high wave number. This is caused by the interaction between BiOBr and CAU‐17, which can further prove the successful preparations of composites.^[25]^ Thermo‐gravimetric was studied from 20 °C to 800 °C in air. CAU‐17 shows a gradual weight loss. There are two steps weight loss before 200 °C (Figure S2). The first step is related to the loss of three H_2_O. The second step is due to the loss of a methanol molecule. The main weight decreases between 20 °C and 380 °C is related to the collapse of MOFs. BiOBr has a huge decline in weight from 630 to 700 °C, corresponds to the decomposition of composite that cause Bi_2_O_3_. As for BCAU‐2 composite, the weight loss between 450 and 630 °C is due to the high temperature decomposition of MOFs.[^30^, ^48^] The weight decreases between 630 to 700 °C, which is caused by the decomposition of BiOBr. Other prepared composites also given for comparison study shown in (Figure S2).

**Figure 2:**
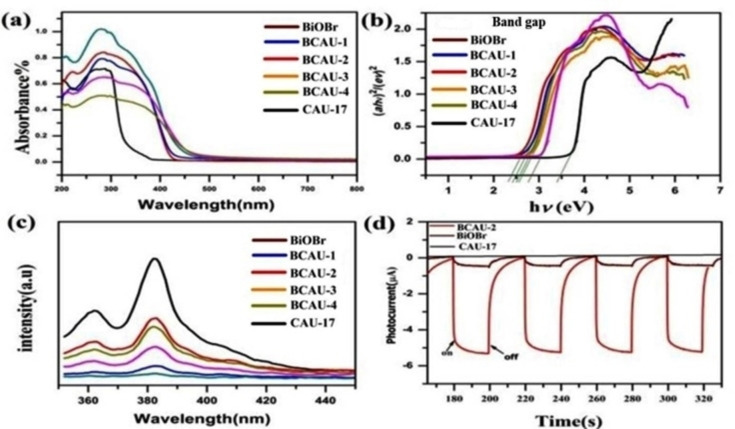
(a) Uv‐vis spectra of BCAU‐X (X=1,2,3,4), BiOBr, and CAU‐17 (b) optical absorption edges (c) Photoluminescence spectra of the as prepared composites with the excitation wavelength of 280 nm, and (d) Transient photo‐current response of BiOBr, BCAU‐2 and CAU‐17.

UV‐vis diffuse reflectance spectra (DRS) of the BCAU‐x (x=1, 2, 3, 4) samples are shown in Figure 2a. The band gap (*Eg*) of BCAU‐2 was estimated about 2.41 E*v* (Figure 2b and Table S5). The band gaps of BiOBr flakes and CAU‐17 were 2.87 E*v* and 3.49 E*v* respectively. The introduction of CAU‐17 with BiOBr can reduces the band gap value and also enhances the ability to absorb visible light and results a better photo‐catalytic activity. Figure 2c shows the photo‐luminescence emission spectra (PL) of CAU‐17, BiOBr and composite BCAU‐x (x=1, 2, 3, 4) samples. The spectra intensity of BCAU‐2 is lower than that of pristine BiOBr and CAU‐17. It indicates that the recombination of photo‐generated carriers of BiOBr can be decreases significantly after compounding CAU‐17 (Figure 2d).^[46]^

**Figure 3:**
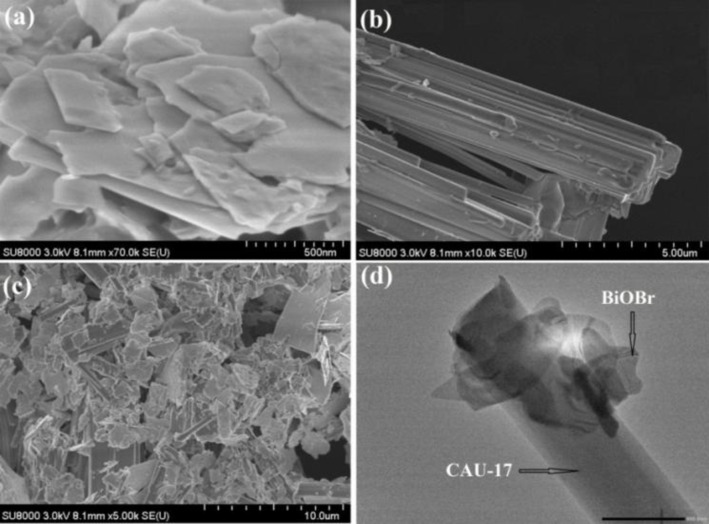
(a) SEM images of BiOBr nanosheet (b) CAU‐17 MOFs (c) SEM image of BCAU‐2 composite and (d) TEM image of BCAU‐2 composite.

To investigate the morphology and structure of the prepared materials, SEM and TEM were performed. The SEM image of BiOBr shows a sheet‐like structure with smooth surfaces (Figure 3a). The SEM image of CAU‐17 shows a rod‐like structure with a smooth surface (Figure 3b). The SEM image of the BCAU‐2 composite shows that BiOBr sheets are distributed uniformly on the surface of the CAU‐17 rods, forming a hierarchical structure (Figure 3c). The TEM images of BiOBr, CAU‐17, and the BCAU‐2 composite are shown in Figure 3a–d, respectively. The TEM image of BiOBr shows a typical layered structure with clear lattice fringes (Figure 3d). The TEM image of CAU‐17 shows a well‐defined rod‐like structure with clear lattice fringes. The TEM image of the BCAU‐2 composite shows that BiOBr sheets are uniformly distributed on the surface of the CAU‐17 rods, and the lattice fringes of BiOBr and CAU‐17 are clearly visible (Figure 3d), which is consistent with the XRD results and further confirms the presence of BiOBr and Bi‐MOFs in the composite. It is evident that the composite has consistently and well‐dispersed BiOBr and Bi‐MOFs.[^22^, ^25^, ^47^]

**Figure 4:**
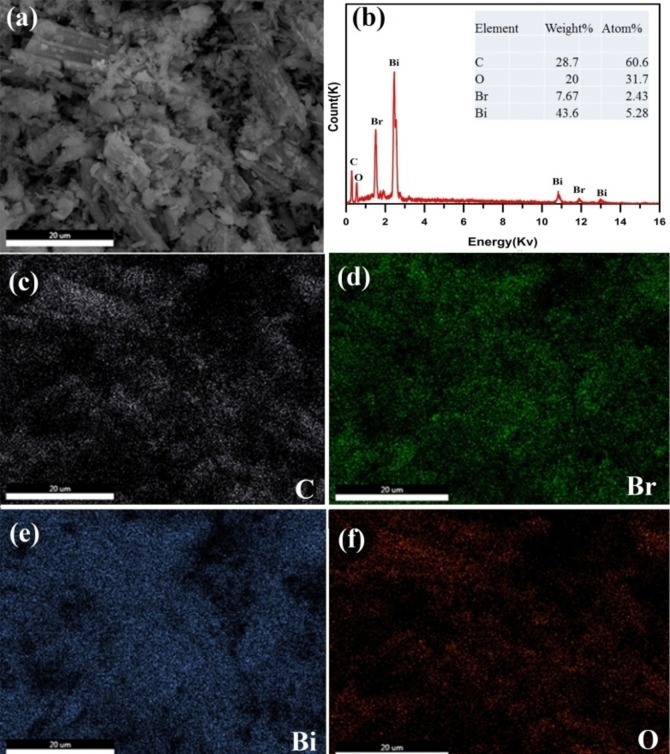
EDS analysis and mapping of BCAU‐2 nano‐composite.

Moreover, in order to prove the above results, EDS analysis and mapping measurements were carried out of the BCAU‐2 composite, indicating that C, O, Br and Bi elements were uniformly distributed in the composite (Figure 4).

**Figure 5:**
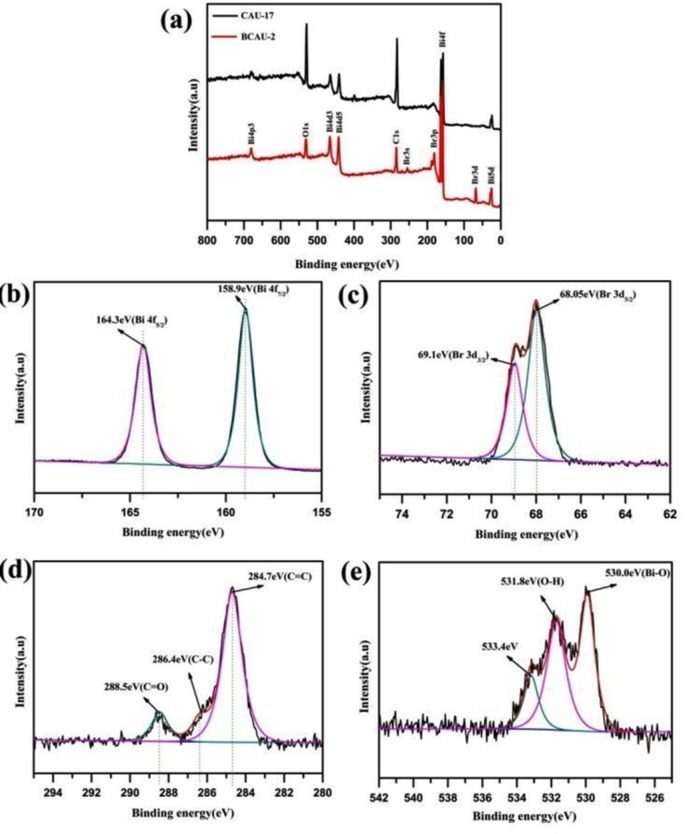
(a) XPS survey scan spectrum of the samples (b) Bi 4f (c) Br 3d (d) C 1s (e) O 1s.

XPS and EDX were used to further investigate the surface chemical composition and element distribution of the prepared materials. The XPS survey spectrum of the BCAU‐2 composite (Figure 5a) shows peaks corresponding to Bi, O, Br, C, and N, indicating the presence of BiOBr and CAU‐17. The high‐resolution XPS spectra of Bi 4 f, O 1s, and Br 3d are shown in Figure 5b–d, respectively. The Bi 4 f spectrum can be fitted into two peaks at 159.4 and 164.4 eV, corresponding to Bi 4f7/2 and Bi 4f5/2, respectively (Figure 5b). The O 1s spectrum can be fitted into two peaks at 530.2 and 532.2 eV. The XPS analysis was carried out to investigate the surface chemical composition of the samples. Figure 5a shows the XPS spectra of Bi 4 f of BiOBr, Bi‐MOFs (CAU‐17), and BCAU‐2 composite. Bi^3+^ in BiOBr is responsible for the peaks at 159.7 eV and 164.1 eV, which correspond to Bi 4f7/2 and Bi 4f5/2, respectively. Bi‐MOFs are responsible for the peaks at 157.1 eV and 161.6 eV (CAU‐17). In Figure 5b displays the O 1s XPS spectra of BiOBr, Bi‐MOFs (CAU‐17), and BCAU‐2 composite. The lattice oxygen is responsible for the peak centered at 530.0 eV, whereas the adsorbed oxygen species is responsible for the peak at 532.4 eV. The adsorption of CO_2_ and H_2_O by oxygen species is responsible for the peak at 533.4 eV. The BCAU‐2 composite contains BiOBr and Bi‐MOFs, as confirmed by the XPS study.[^41^, ^45^, ^58^]

### Photo‐Catalytic Properties

**Figure 6:**
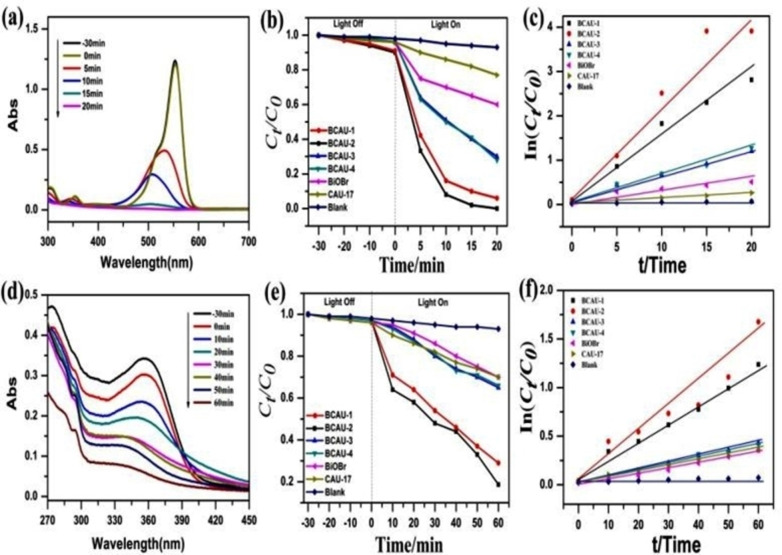
(a) Time dependent UV‐vis absorption spectra for RhB degradation by BCAU‐2 (b) Photo‐catalytic degradation efficiencies of RhB with different prepared samples under visible light irradiation (c) comparisons of the rate constants *k* (pseudo‐first order kinetic model) in the presence of different prepared samples (d) time dependent Uv‐vis absorption spectra for TC degradation by bCAU‐2 (e) photo‐catalytic degradation efficiencies of TC with different prepared samples under visible light irradiation (f) comparison of the rate constant k (pseudo‐first order kinetic model) in the presence of different prepared samples.

The photo‐catalytic activity of the samples was evaluated by the degradation of RhB and TC under visible light irradiation. As shown in Figure 6a, the BiOBr/CAU‐17 composite exhibits much higher photo‐catalytic activity than BiOBr and Bi‐MOFs (CAU‐17) as shown in Figure 6b. Among the BiOBr/CAU‐17 composites, BCAU‐2 exhibits the highest photo‐catalytic activity towards RhB degradation. The pseudo‐first‐order rate constant (k) for RhB degradation over BiOBr/CAU‐17 is calculated to be 0.083 min−1, which is much higher than that of BiOBr (0.036 min−1) and Bi‐MOFs (CAU‐17) (0.031 min−1).^[48]^ The high photo‐catalytic activity of BCAU‐2 can be attributed to the synergistic effect between BiOBr and Bi‐MOFs. The efficient transfer of photo‐generated carriers at the interface between BiOBr and Bi‐MOFs can improve the separation efficiency of photogenerated electron‐hole pairs, thus promoting the photo‐catalytic activity. In addition, the high specific surface area of Bi‐MOFs can provide more active sites for the adsorption of organic pollutants, which can further enhance the photo‐catalytic activity.^[45]^

The photo‐catalytic mechanism of RhB dye and TC degradation over BiOBr/CAU‐17 was studied by capturing active species. As shown in Figure 6b, the degradation efficiency of RhB decreases significantly in the presence surface area and pore volume of BiOBr, CAU‐17, and the BCAU‐2 composite were measured by N_2_ adsorption/desorption isotherms. As shown in Figure 6a, the isotherm of BiOBr exhibits type IV with H_2_‐type hysteresis loops, indicating that BiOBr has mesoporous structure 47. The isotherm of CAU‐17 exhibits type I with a sharp increase in the relative pressure region of 0.8–1.0, indicating a microporous structure. The BCAU‐2 composite exhibits both the features of BiOBr and CAU‐17, indicating the successful preparation of the composite. The BET specific surface area and pore size distribution of BiOBr, CAU‐17, and the BCAU‐2 composite are shown in Figure 6b. The BET surface area of the BCAU‐2 composite (91.2 m2/g) is higher than that of BiOBr (23.6 m2/g), which indicates that the addition of MOFs effectively enhances the specific surface area of the composite.[^12^, ^31^] The pore size distribution curve of BiOBr exhibits a single peak centered at 6.46 nm, while that of CAU‐17 exhibits a peak centered at 1.63 nm, indicating the presence of micropores. The BCAU‐2 composite exhibits a bimodal pore size distribution curve with the peaks at 1.63 nm and 5.95 nm, indicating that the composite has both micropores and mesopores.^[49]^

**Scheme 1:**
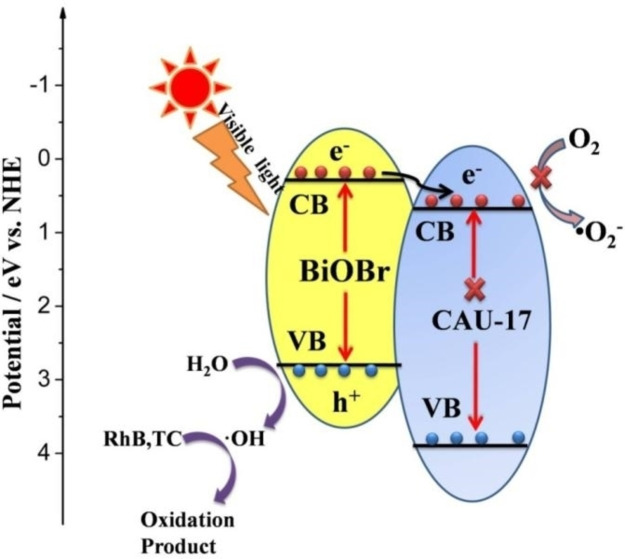
Possible Photo‐catalytic Degradation Mechanism under Visible Light by BCAU‐2 composite (a) RhB and (b) TC.

Catalyst stability is a key factor in practical application view point. As shown in Figure S3(a) RhB and (b) TC, BCAU‐2 composite has good photo‐stability, apparently no obvious decrease after three consecutive catalytic cycles. The excellent activity and stability of BCAU‐2 material make it become a promising photo‐catalytic material used in the degradation of different color dyes and colorless organic pollutants. The possible degradation mechanism of RhB and TC was discussed and shown in Scheme 1. It was predominantly formed of Bi 6p orbitals and mostly composed of O 2p orbitals with additions from Bi 6 s orbitals. With a band gap of roughly 2.7 eV, BiOBr is visible light‐responsive. The photons are absorbed by the electrons in the valence band of BiOBr and stimulated to the conduction band when it is exposed to light with energy equal to or greater than its band gap, which is approximately 2.7 eV. In the conduction band, this excitation creates free electrons and leaves holes in the valence band. In photocatalysis, electron‐hole pairs are the main active species. Increased surface area, better charge separation, and prolonged light absorption are the advantages of the BiOBr/CAU‐17 composite, which boosts photocatalytic activity for uses such as environmental remediation and pollutant degradation.[^14^, ^24^] The separated electrons and holes can react with adsorbed O_2_/H_2_O and produced highly active free radicals, which can degrade the dyes. The *E_g_* of BiOBr and CAU‐17 is 2.87 eV and 3.49 eV, respectively (Table. S5). Therefore, only BiOBr can absorb visible light and produce photo‐excited carriers. According to the reference data, the band edge of CB potential of BiOBr is 0.27 eV, which is positive than the O_2_/O_2_^−^ potential (−0.046 V vs NHE). Therefore, the electrons on the CB of BiOBr cannot generate ⋅O_2_^–^ radical. The photo‐generated electrons move to the CB of CAU‐17, which can efficiently reduce the rate of carriers’ recombination and enhance the photo‐catalysis performance. The holes (h^+^) can further react with H_2_O to produce ⋅OH radical. The RhB and TC molecules can be oxidized by the h^+^ and ⋅OH species.[^35^, ^38^, ^45^]

### Nitrogen Adsorption‐Desorption Isotherm

**Figure 7:**
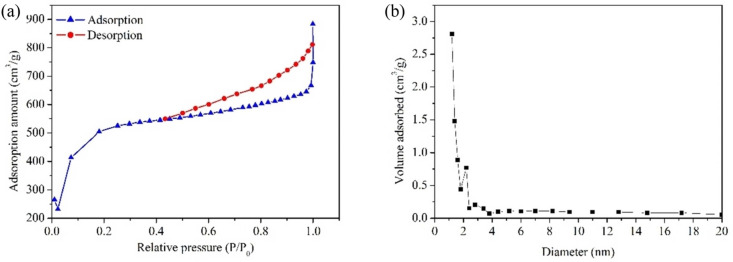
(a) N_2_ adsorption‐desorption isotherms and (b) pore size distribution curve based on the Brunauer‐Emmett‐Teller method for the activated carbon.

An essential characterization tool for materials such as the BiOBr/CAU‐17 composite is the nitrogen (N_2_) adsorption/desorption isotherm, which is used to analyze the surface area, pore size distribution, and porosity. The textural properties of the composite are influenced by these isotherms and have a direct bearing on its performance in applications such as photocatalysis. In Figure 7a **isotherm** indicates a microporous material with a high affinity for nitrogen at low relative pressures (P/P₀<0.1). In **Type IV Isotherm**, suggests the presence of mesopores, characterized by a hysteresis loop at medium to high relative pressures (P/P₀=0.4–0.9). The Brunauer‐Emmett‐Teller (BET) surface area of the BiOBr/CAU‐17 composite is calculated from the linear portion of the isotherm (typically at relative pressures P/P₀ between 0.05 and 0.3) Figure 7b. A high BET surface area indicates a large available surface for catalytic reactions, which is beneficial for photocatalysis. The composite might exhibit a BET surface area significantly higher than that of pure BiOBr, due to the contribution of CAU‐17′s porous structure.[^45^, ^49^] The textural properties derived from N₂ adsorption/desorption isotherms are directly related to the photocatalytic performance of the BiOBr/CAU‐17 composite: the N_2_ adsorption/desorption isotherms of the BiOBr/CAU‐17 composite provide critical information about its surface and pore characteristics, which are essential for optimizing and understanding its photocatalytic behavior.^[54]^

## Conclusions

In conclusion, a series of BiOBr/CAU‐17 nanostructures with different MOFs (CAU‐17) content were prepared by simple co‐precipitation method. The prepared nano‐composites are simple, economical and easy to prepare. The loading amount of MOFs (CAU‐17) can regulate the photo‐catalytic performance of the composites. Among them, BCAU‐2 nano‐composite has excellent visible light photo‐catalytic performance, which is 9.9 and 20 times higher than that of pristine BiOBr and CAU‐17, respectively for the degradation of RhB and 8.4 and 17 times higher for the degradation of TC. Furthermore, the active species trapping experiments revealed that the hole^+^ and.O_2_^−^ radicals play a major role in photo‐catalytic degradation of RhB and TC. The improved photo‐catalytic performance of BCAU‐2composite can be attributed to fast interfacial charge transfer. The photocatalytic degradation of TC was significantly enhanced by the BiOBr bimetal modification, with the 2 wt.% BiOBr/CAU‐17 nanocomposite achieving an 87.2 % degradation of TC and 82 % Total Organic Carbon (TOC) removal within 60 min. The BiOBr/CAU‐17 composite represents a significant advancement in photocatalytic materials, offering enhanced performance for the degradation of dyes and the removal of tetracycline from water. Its combination of efficient light absorption, high surface area, and excellent charge separation makes it an ideal candidate for environmental remediation applications, particularly in addressing water pollution challenges. Continued research and optimization could further improve its effectiveness and scalability for industrial use. We are confident that this finding will open up new avenues for the development of simple, convenient and inexpensive photo‐catalyst materials to keep the aquatic environment clean and safe.

## Author Contributions

Mansoor Akhtar and Shifa Ullah Khan proposed the idea for work. Mansoor Akhtar and Muhammad Ahmad conducted the synthesis and characterization analysis. Tansir Ahamad and Ghulam Mustafa processed the data and polished the manuscript. Shifa Ullah Khan supervised the project.

## Conflict of Interests

The authors declare that they have no known competing financial interests or personal relationships that could have appeared to influence the work reported in this paper.

1

## Supporting information

As a service to our authors and readers, this journal provides supporting information supplied by the authors. Such materials are peer reviewed and may be re‐organized for online delivery, but are not copy‐edited or typeset. Technical support issues arising from supporting information (other than missing files) should be addressed to the authors.

Supporting Information

## Data Availability

The data that support the findings of this study are available from the corresponding author upon reasonable request.
