# Supplementary material for: A Novel BiOBr/CAU‐17 Composite with Enhanced Photo‐Catalytic Performance for Dye Degradation and Removal of Tetracycline Antibiotic Under Visible Light
Source: ChemistryOpen. 2024 Oct 23;14(1):e202400195. doi: 10.1002/open.202400195 (PMC11726658; doi:10.1002/open.202400195)
Supplement: Supplementary file 1 — Supporting Information [file OPEN-14-e202400195-s001.pdf]

ChemistryOpen

Supporting Information

# A Novel BiOBr/CAU-17 Composite with Enhanced Photo-Catalytic Performance for Dye Degradation and Removal of Tetracycline Antibiotic Under Visible Light

Mansoor Akhtar,* Shifa Ullah Khan,* Ghulam Mustafa, Muhammad Ahmad, and Tansir Ahamad

# Electronic Supplementary Information

## A Novel BiOBr/CAU-17 Composite with Enhanced Photo-catalytic Performance for Dye Degradation and Removal of Tetracycline Antibiotic under Visible Light

Mansoor Akhtar*[a], Shifa Ullah Khan*[b], Ghulam Mustafa [b], Muhammad Ahmad [a], Tansir Ahamad [c]

[a] College of Civil and Transportation Engineering, Shenzhen University, Shenzhen, Guangdong, 518060, P, R, China, Institute for Advanced Study, Shenzhen University, Shenzhen, Guangdong 518060, P. R. China.

[b] The institute of Chemistry, Faculty of Science, University of Okara, Renala Campus 56100, Punjab, Pakistan.

[c] Department of Chemistry, College of Science, King Saud University, Riyadh 11451, Saudi Arabia

Correspondence:

Dr. Mansoor Akhtar akt100@nenu.edu.cn  Dr. Shifa Ullah Khan kee100@nenu.edu.cn

## 1. Experimental

### 1.1. Materials and syntheses

Bismuth nitrate pentahydrate, 1, 3, 5 Trimesic Acid, sodium Bromide, RhB and tetracycline hydrochloride were supplied by Macklin Shanghai China. All reagents for synthesis and analysis were used without further purification. All of the reaction solutions were prepared using deionized water. CAU-17 MOF was synthesized as reported[25].

### 1.2. Preparation of BiOBr/CAU-17 composites

BiOBr/CAU-17 composites were prepared through a simple solution method. FourBiOBr/CAU-17composites named as (BCAU-1, BCAU-2, BCAU-3, and BCAU-4) with different MOF contents value (CAU-17 MOF= 0.12g, 0.17g, 0.3g and 0.7g respectively) were prepared. Typically 0.145 g of NaBr was dissolved in 10 ml D.I water then calculated amount of CAU-17 MOF was added, and the mixture was kept stirring vigorously for 2 h. Afterwards 0.242 g Bi $(NO_3)_3.5H_2O$ was dissolved in 5 mL DMF, and this Bi $(NO_3)_3$ DMF solution was added to the CAU-17 mixture drop wise with continuous stirring. The mixture was kept undisturbed for 1 hour at room temperature. Finally, the product was collected by filtration, marked as BCAU-1, BCAU-2, BCAU-3, and BCAU-4 respectively and extensively washed with deionized water for several times, dried in vacuum oven at 80°C for further use. Pristine BiOBr was also synthesized by the same procedure without adding CAU-17 MOF.

### 1.3. Characterization

X-ray diffraction (XRD) patterns of samples were scanned on the Bruker AXS D8 Focus using filtered Cu-Kα radiation (λ= 1.54056 Å). The FTIR spectra were recorded from KBr pellets in therange 4000-400 cm-1 on an Alpha Centaur FT/IR spectrometer. UV−vis diffuse reflectance spectra (DRS) of the samples were collected on a UV-2600 UVvis spectrophotometer (Shimadzu), with an integrating sphere, and BaSO4 was used as the reference. A JEOL JSM 4800F SEM coupled with an energy-dispersive X-ray (EDS) spectrometer was used to characterize sample morphology. High-resolution TEM (transmission electron microscopy) images were obtained via an FEI Tecnai G2 operated at 200 kV. XPS were carried out on an ESCALABMKII spectrometer with an Al-Ka (1486.6 eV) achromatic X-ray source.

### 1.4. Evaluation of the photo-catalytic activity

The photo-catalytic activities of the as-prepared samples were evaluated by degradation of Rhodhamine B (RhB) solution and Tetracycline (TC) antibiotic using a 300 W Xe lamp (Peking ceaulight, CEL-HXF300) with a 420 nm cut-off filter as the light source and a self-made glass

vessel with a water-cooling jacket as reactor. The irradiation distance between the lamp and the mixture solution was 15 cm. For the degradation experiment with BCAU-1, BCAU-2, BCAU-3, and BCAU-4, 20mg of photo-catalyst were dispersed in 50 mL 20 ppm of RhB and TC solutions. Prior to irradiation, the suspensions were magnetically stirred for 30 min in the dark to ensure that the adsorption– desorption equilibrium between the organic molecules and the catalyst surface was reached. During the entire process of dye degradation and TC solution, the solid suspension was under magnetic stirring. At certain time intervals, 2 mL aliquots were sampled and centrifuged and measured on a XinMao UV-vis spectrometer UV-7502. For the degradation experiment with BCAU-1, BCAU-2, BCAU-3, and BCAU-4, as well as the control experiments, all conditions were samefor the entire photo-catalytic experiments. The blank experiment without any catalyst, Pristine BiOBr and CAU-17 MOF were also examined to compare the effectiveness with prepared hybrid materials.

**1.5 Photocurrent measurements**

To investigate the transition of photo-generated electrons of the BiOBr, CAU-17 and BCAU-2 composite, the photo-current was measured with an electrochemical analyzer (CHI660E, Chenhua, Shanghai, China) in a standard three electrode system with the samples as the working electrode, a Pt foil as the counter electrode, and a saturated calomel electrode (SCE) as a reference electrode. The working electrode was prepared by making a suspension, which was then dropped onto a 1 cm ×1 cm ITO glass electrode. A 300 W Xe lamp (Shanghai BILON Instrument Corporation) was utilized as a light source. 0.2 M of $Na_2SO_4$ aqueous solution was used as the supporting electrolyte.

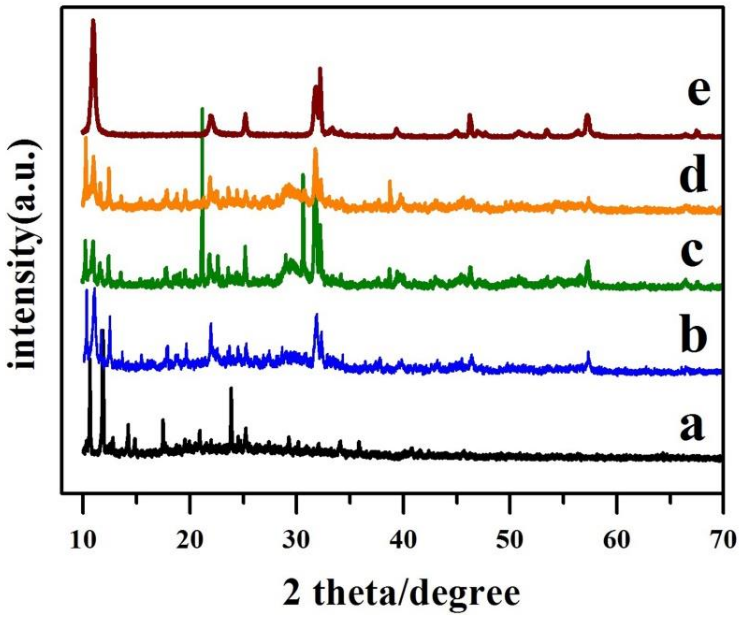


Fig.S1. XRD patterns of CAU-17(a) BCAU-1 (b), BCAU-3(c), BCAU-4(d) composites,and (e) pristine BiOBr for comparison.

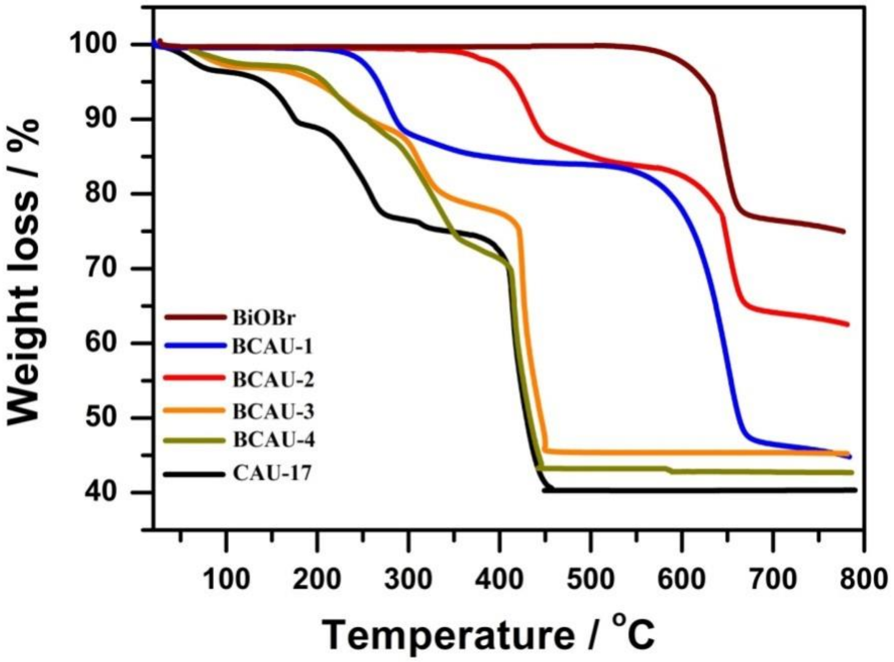


Fig.S2. Thermal decomposition patterns of BiOBr, CAU-17 and BCAU-x (BCAU-1,BCAU-2,BCAU-3 and BCAU-4) composites under oxygen atmosphere.

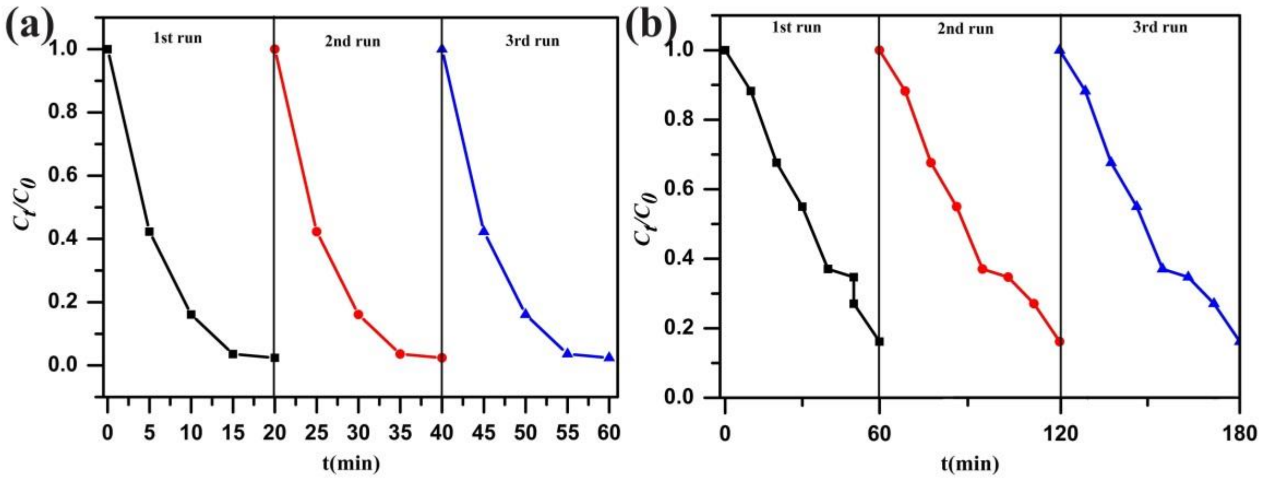


Fig.S3.(a) Recycling test of BCAU-2 for the degradation ofRhB and (b) TC under visible-light irradiation.

## 1.6. Investigation of the photo-catalytic mechanism

To investigate the photocatalytic mechanism of the photocatalyst, isopropyl alcohol (IPA), benzoquinone (BQ), and triethanolamine (TEOA) were introduced as scavengers for hydroxyl radicals(•OH),superoxide radicals ($.O^{2-}$), and holes ($h^+$), respectively. Radical trapping experiments were further conducted to estimate the reactive active species generated during the irradiation of the BCAU-2 sample. During the experiments, IPA, BQ, or TEOA were added to the RhB/TC solution to give concentrations of 1 mM, and all other conditions remained the same as those used in the afore mentioned degradation of RhB/TC experiments.

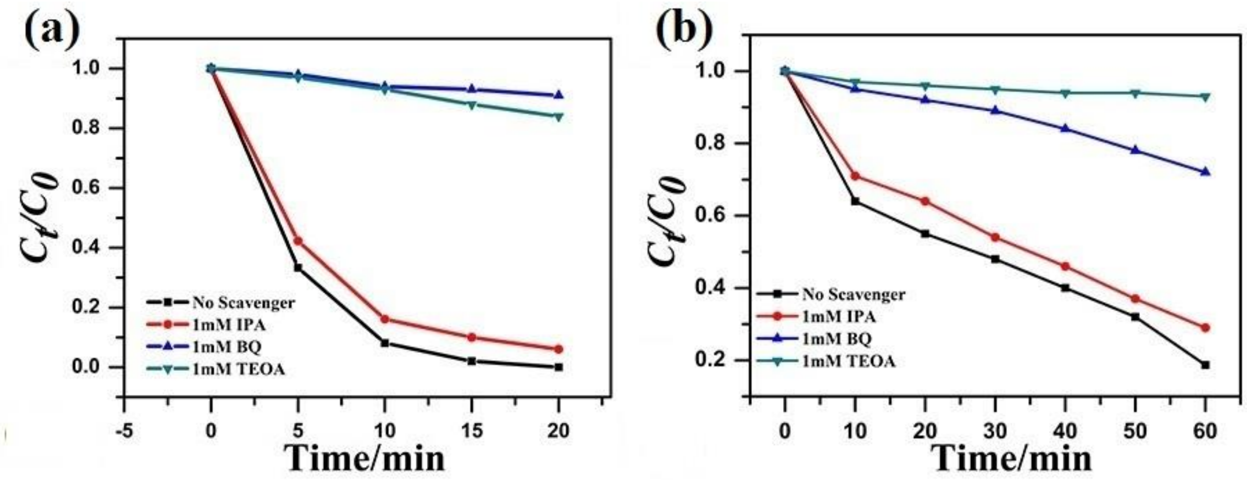


Fig.S4.(a) Effect of scavengers on the photo-catalytic degradation of RhB by BCAU-2 and (b) Effect of scavengers on the photo-catalytic degradation of TC by BCAU-2.

**Table.S1.**Pseudo-first-order rate constant for RhB photo-catalytic degradation under BiOBr/CAU-17-x system (x=1, 2, 3 and 4).

| **Entry** | **Photo-catalyst** | **First-order kinetic equation** | **k ($min^{-1}$)** |
|---|---|---|---|
| **1** | CAU-17 | 0.0094 $min^{-1}t$ | 0.0094 |
| **2** | BiOBr | 0.0190 $min^{-1}t$ | 0.0190 |
| **3** | BCAU-1 | 0.1455$min^{-1}t$ | 0.1455 |
| **4** | BCAU-2 | 0.1882 $min^{-1}t$ | 0.1882 |
| **5** | BCAU-3 | 0.0597 $min^{-1}t$ | 0.0597 |
| **6** | BCAU-4 | 0.0539 $min^{-1}t$ | 0.0539 |

**Table.S2.**Pseudo-first-order rate constant for TC photo-catalytic degradation under BiOBr/CAU-17-x system (x=1, 2, 3 and 4).

| **Entry** | **Photo-catalyst** | **First-order kinetic equation** | **k ($min^{-1}$)** |
|---|---|---|---|
| **1** | CAU-17 | 0.0061$min^{-1}t$ | 0.0061 |
| **2** | BiOBr | 0.0043$min^{-1}t$ | 0.0043 |
| **3** | BCAU-1 | 0.0186$min^{-1}$ $t$ | 0.0186 |
| **4** | BCAU-2 | 0.0200$min^{-1}$ $t$ | 0.0200 |
| **5** | BCAU-3 | 0.0065$min^{-1}t$ | 0.0065 |
| **6** | BCAU-4 | 0.0060$min^{-1}$ $t$ | 0.0060 |

**Table.S3.**Summary of the photo-degradation performance of reported BiOBr/MOFs for RhB pollutants.

| Composite | Irradiation source | Organic pollutants | Initial concentration (mg $L^{-1}$) | Time (min) | Sample amount (mg) | Solution volume (ml) | Ref. |
|---|---|---|---|---|---|---|---|
| **BiOBr/Uio-66** | sunlight | RhB | 14.4 | 15 | 15 | 30 | 1 |
| **BiOBr/$NH_2$-MIL-125(Ti)** | sunlight | RhB | 20 | 100 | 40 | 200 | 2 |
| **BiOBr/CAU-17-2h*in-situ*** | sunlight | RhB | 20 | 50 | 40 | 200 | 3 |
| **BiOBr/Uio-66-$NH_2$** | sunlight | RhB | 10 | 120 | 40 | 250 | 4 |
| **BiOBr/CAU-17** | sunlight | RhB | 20 | 20 | 20 | 50 | This work |

**Table.S4.**Summary of the photo-degradation performance of some reported different photo-catalysts for TC pollutants.

| Composite | Irradiation source | Organic pollutants | Initial concentration (mg $L^{-1}$) | Time (min) | Sample amount (mg) | Solution volume (ml) | Ref. |
|---|---|---|---|---|---|---|---|
| **BiOI** | sunlight | TC | 40 | 240 | 50 | 50 | 5 |
| **BiOBr/BN** | sunlight | TC | 20 | 80 | 50 | 100 | 6 |
| **Bi24O31Br10** | sunlight | TC | 20 | 90 | 10 | 30 | 7 |
| **Ag3VO4/BiOBr** | sunlight | TC | 10 | 60 | 30 | 50 | 8 |
| **CQDs/BiOBr** | sunlight | TC | 20 | 80 | 50 | 100 | 9 |
| **Au/Pt/g-C3N4** | sunlight | TC | 20 | 180 | 100 | 100 | 10 |
| **BiOBr/CTF** | sunlight | TC | 10 | 50 | 40 | 200 | 11 |
| **Ag/Bi3TaO7** | sunlight | TC | 10 | 60 | 50 | 1000 | 12 |
| **AgI/BiVO4** | sunlight | TC | 20 | 60 | 30 | 100 | 13 |
| **BiOBr/CAU-17** | sunlight | TC | 20 | 60 | 20 | 50 | This work |

**Table.S5.** Summary of the optical absorption band gap energies of different prepared samples and starting materials.

| Entry | Band gap energy |
|---|---|
| **BCAU-1** | **2.60** |
| **BCAU-2** | **2.41** |
| **BCAU-3** | **2.70** |
| **BCAU-4** | **2.73** |
| **BiOBr** | **2.87** |
| **CAU-17 MOF** | **3.49** |

The HOMO–LUMO energy band gap of BCAU-x (x=1, 2, 3, 4) can be estimated from the Tauc's plots using $\alpha\ (h\nu) = A\ (h\nu\text{-}Eg)^{n/2}$, where α, *h*, *v*, $E_g$, and A are the absorption coefficient, Plank's constant, light frequency, band gap energy, and a constant, respectively. The coefficient 'n' is related to the optical transition of the semiconductors (n=1 for direct transition and n=4 for indirect transition).

## References


1- Sha, Zhou, and Jishan Wu. "Enhanced visible-light photocatalytic performance of BiOBr/UiO-66 (Zr) composite for dye degradation with the assistance of UiO-66." *Rsc Advances* 5, no. 49 (2015): 39592-39600.

2- Zhu, Shuai-Ru, Peng-Fei Liu, Meng-Ke Wu, Wen-Na Zhao, Guo-Chang Li, Kai Tao, Fei-Yan Yi, and Lei Han. "Enhanced photocatalytic performance of BiOBr/NH 2-MIL-125 (Ti) composite for dye degradation under visible light." *Dalton Transactions* 45, no. 43 (2016): 17521-17529.

3- Zhu, Shuai-Ru, Meng-Ke Wu, Wen-Na Zhao, Peng-Fei Liu, Fei-Yan Yi, Guo-Chang Li, Kai Tao, and Lei Han. "In situ growth of metal–organic framework on BiOBr 2D material with excellent photocatalytic activity for dye degradation." *Crystal Growth & Design* 17, no. 5 (2017): 2309-2313.

4- Bibi, Rehana, Quanhao Shen, Lingfei Wei, Dandan Hao, Naixu Li, and Jiancheng Zhou. "Hybrid BiOBr/UiO-66-NH 2 composite with enhanced visible-light driven photocatalytic activity toward RhB dye degradation." *RSC advances* 8, no. 4 (2018): 2048-2058.

5- Hao, Rong, Xin Xiao, Xiaoxi Zuo, Junmin Nan, and Weide Zhang. "Efficient adsorption and visible-light photocatalytic degradation of tetracycline hydrochloride using mesoporous BiOI microspheres." *Journal of hazardous materials* 209 (2012): 137-145.

6- Di, Jun, Jiexiang Xia, Mengxia Ji, Bin Wang, Sheng Yin, Qi Zhang, Zhigang Chen, and Huaming Li. "Advanced photocatalytic performance of graphene-like BN modified BiOBr flower-like materials for the removal of pollutants and mechanism insight." *Applied Catalysis B: Environmental* 183 (2016): 254-262.

7- Wang, Chu-Ya, Xing Zhang, Hai-Bin Qiu, Gui-Xiang Huang, and Han-Qing Yu. "$Bi_{24}O_{31}Br_{10}$ nanosheets with controllable thickness for visible–light–driven catalytic degradation of tetracycline hydrochloride." *Applied Catalysis B: Environmental* 205 (2017): 615-623.

8- Zhang, Junlei, and Zhen Ma. "Flower-like $Ag_3VO_4$/BiOBr np heterojunction photocatalysts with enhanced visible-light-driven catalytic activity." *Molecular Catalysis* 436 (2017): 190-198.

9- Di, Jun, Jiexiang Xia, Mengxia Ji, Bin Wang, Xiaowei Li, Qi Zhang, Zhigang Chen, and Huaming Li. "Nitrogen-doped carbon quantum dots/BiOBr ultrathin nanosheets: In situ strong coupling and improved molecular oxygen activation ability under visible light irradiation." *ACS Sustainable Chemistry & Engineering* 4, no. 1 (2016): 136-146.

10- Xue, Jinjuan, Shuaishuai Ma, Yuming Zhou, Zewu Zhang, and Man He. "Facile photochemical synthesis of Au/Pt/g-$C_3N_4$ with plasmon-enhanced photocatalytic activity for antibiotic degradation." *ACS applied materials & interfaces* 7, no. 18 (2015): 9630-9637.

11- Zhu, Shuai-Ru, Qi Qi, Yuan Fang, Wen-Na Zhao, Meng-Ke Wu, and Lei Han. "Covalent triazine framework modified BiOBr nanoflake with enhanced photocatalytic activity for antibiotic removal." *Crystal Growth & Design* 18, no. 2 (2018): 883-891.

12- Luo, Bifu, Dongbo Xu, Di Li, Guoling Wu, Miaomiao Wu, Weidong Shi, and Min Chen. "Fabrication of a Ag/$Bi_3TaO_7$ plasmonic photocatalyst with enhanced photocatalytic activity for degradation of tetracycline." *ACS applied materials & interfaces* 7, no. 31 (2015): 17061-17069.

13- Chen, Fei, Qi Yang, Jian Sun, Fubing Yao, Shana Wang, Yali Wang, Xiaolin Wang et al. "Enhanced photocatalytic degradation of tetracycline by AgI/$BiVO_4$ heterojunction under visible-light irradiation: mineralization efficiency and mechanism." *ACS applied materials & interfaces* 8, no. 48 (2016): 32887-32900.
